# Acute Ischemic Stroke: A Retrospective Study Comparing Clinical Characteristics and Outcomes in Patients With and Without Complications

**DOI:** 10.7759/cureus.103902

**Published:** 2026-02-19

**Authors:** Anida Abazovic Bihorac, Mirza Kovacevic

**Affiliations:** 1 Neurology, Cantonal Hospital Zenica, Zenica, BIH; 2 Faculty of Medicine, University of Zenica, Zenica, BIH; 3 Anesthesia and Critical Care, Cantonal Hospital Zenica, Zenica, BIH

**Keywords:** acute ischemic stroke, complications’, hospital outcomes, nihss score, post-stroke complications

## Abstract

Background: Acute ischemic stroke (AIS) is a leading cause of morbidity and mortality. Post-stroke complications, both neurological and systemic, negatively affect patient outcomes, prolong hospitalization, and increase healthcare costs. Identifying high-risk patients is essential for early intervention.

Aim: To compare clinical, radiological, laboratory characteristics, and in-hospital outcomes between patients with AIS who developed complications and those who did not.

Methods: This retrospective cohort study included 150 patients with confirmed first AIS admitted between October 2023 and October 2024. Patients were divided into two groups: Group 1 (*n* = 73) with in-hospital complications and Group 2 (*n* = 77) without complications. Demographic data, comorbidities, National Institutes of Health Stroke Scale (NIHSS) scores, brain computer tomography (CT) findings, laboratory parameters, blood pressure, complications, and outcomes were analysed. Continuous variables are presented as median (interquartile range) and categorical variables as number (%). A *P*-value < 0.05 was considered statistically significant.

Results: Group 1 patients were older (73.0 (interquartile range (IQR) 66.5-79.0) vs. 69.0 (IQR 62.0-73.0) years; *P* < 0.001) and had higher NIHSS scores at admission (10.0 (IQR 5.0-16.0) vs. 5.0 (IQR 4.0-7.0); *P* < 0.001) and follow-up (6.0 (IQR 4.0-11.0) vs. 3.0 (IQR 2.0-5.0); *P* < 0.001). Large infarctions were more frequent in Group 1 (57.5% vs. 27.3%; *P* < 0.001), and glucose levels were higher (14.0 (IQR 10.1-16.3) vs. 6.8 (IQR 5.95-9.65) mmol/L; p = 0.027). Length of hospital stay and in-hospital mortality were greater in Group 1 (14.0 (IQR 10.0-17.0) vs. 7.0 (IQR 6.0-10.0) days; *P* < 0.001; 17.8% vs. 3.9%, respectively).

Conclusions: Patients with AIS who develop complications have distinct clinical and laboratory profiles, more severe neurological deficits, and worse in-hospital outcomes. Early risk identification may improve management and patient care.

## Introduction

Acute ischemic stroke (AIS) is one of the leading causes of morbidity and mortality worldwide, representing a major burden on healthcare systems and society. Each year, millions of individuals experience an AIS event, and despite significant advances in acute management, including reperfusion therapies and standardized supportive care, a substantial proportion of patients still develop complications during hospitalization [1]. These complications may have profound effects on patient outcomes, prolong hospital stay, increase healthcare costs, and contribute to long-term disability or death [2].

Post-stroke complications can be broadly classified into neurological and systemic events. Neurological complications may include dysphagia, epileptic seizures, and deterioration of neurological function, while systemic complications commonly comprise urinary tract infections, pneumonia, deep vein thrombosis, renal dysfunction, pulmonary embolism, and pressure ulcers [3]. The occurrence of such complications is often associated with advanced age, preexisting comorbidities, stroke severity, and various clinical or laboratory parameters at admission [4].

Understanding which patients are at higher risk is crucial for optimizing monitoring, tailoring interventions, and ultimately improving clinical outcomes. Several studies have examined individual risk factors for post-stroke complications, such as infection or venous thromboembolism [5-7], and some have evaluated predictive scoring systems for the early identification of high-risk patients [8]. However, comprehensive data systematically comparing the clinical, radiological, and laboratory characteristics of patients who develop complications with those who do not remain limited. Previous studies have primarily focused on individual complications, such as dysphagia, whereas comprehensive evaluations integrating laboratory inflammatory markers, microbiological findings, and clinical outcomes during the same hospitalization remain limited.

This retrospective observational study aims to address this gap by comparing characteristics, radiological findings, laboratory parameters, and in-hospital outcomes between patients with AIS who developed specific complications, such as respiratory, infectious, or procedural events, and those who did not. Key confounding factors, including age, comorbidities, stroke severity, and treatment, were taken into account to better understand factors associated with the development of complications.

The primary objective of this study was to identify admission predictors (clinical, imaging, and laboratory parameters) associated with the development of in-hospital complications after a first AIS. Secondary objectives included comparing the length of hospital stay, discharge disposition, and in-hospital mortality between patients with and without complications.

## Materials and methods

Patients

This single-center retrospective cohort observational study was conducted over one year, from October 2023 to October 2024, at the Department of Neurology, Cantonal Hospital Zenica, Bosnia and Herzegovina, after approval by the local Ethics Committee. During the study period, 150 patients were admitted with a diagnosis of AIS. Following approval by the institutional Ethics Committee, all 150 adult patients who met the study criteria were included.

Inclusion criteria comprised all patients admitted with confirmed first AIS based on diagnostic criteria described by Yew et al. [9].

Exclusion criteria were age <18 years, previous history of AIS, head trauma, or other central nervous system diseases, including intracerebral hemorrhage, brain tumors, neurodegenerative disorders, demyelinating diseases, and active central nervous system infections.

Group classification

Patients were divided into two groups according to the presence of complications during hospitalization. Group 1 included patients who developed any of the following complications: cerebral (dysphagia, epileptic seizures, and deterioration of neurological function) or extracerebral/systemic (psychiatric disturbances [any acute change in mental status], urinary incontinence, renal insufficiency, pulmonary embolism, deep venous thrombosis, urinary tract infections, and pressure ulcers). Group 2 included patients who did not develop any of the above complications during hospitalization.

Methods

AIS was defined as an acute episode of focal neurological dysfunction persisting for more than 24 hours, confirmed by imaging evidence of ischemia on computed tomography (CT) [10]. All patients were managed according to the standard diagnostic and therapeutic protocol for AIS [11]. Treatment followed standard post-stroke care and included antiplatelet therapy, statin use, and blood pressure management according to institutional protocols. Intravenous thrombolysis or endovascular thrombectomy was performed when indicated. Additional therapies, such as corticosteroids, proton pump inhibitors, or vitamin supplementation, were administered selectively based on individual patient needs.

Data collection

The following data were collected at admission: demographic characteristics (age and gender), comorbidities, and neurological status assessed using the NIHSS at two time points (T1, admission; T2, transfer or discharge). Ultrasound Doppler examination included assessment of carotid artery stenosis (degree of stenosis) and vertebral artery pathology. 

Stroke classification was based on non-contrast CT findings. Minor stroke was defined as a small cortical or subcortical infarction without territorial involvement, while major stroke was defined as an infarction involving a large vascular territory (e.g., ≥1/3 of the middle cerebral artery territory) or multilobar involvement. Hemorrhagic transformation was classified according to radiological evidence on follow-up CT and categorized as present or absent.

Complications were predefined and classified as cerebral or extracerebral (systemic). Cerebral complications included hemorrhagic transformation (yes/no) and neurological deterioration, such as dysphagia or epileptic seizures. Extracerebral complications were defined as clinically relevant events that were reliably recorded in our patient cohort. These included: urinary tract infection - defined as the presence of urinary symptoms and/or fever with positive urinalysis findings and/or a positive urine culture requiring antibiotic treatment; deep venous thrombosis confirmed by compression Doppler ultrasonography performed upon clinical suspicion; pulmonary embolism - diagnosed using CT pulmonary angiography in symptomatic patients; psychiatric disturbances - defined as acute changes in mental status occurring during hospitalization. Delirium was assessed using the Confusion Assessment Method. Agitation severity was evaluated using the Richmond Agitation-Sedation Scale [12]. Depressive symptoms were screened using the Hospital Anxiety and Depression Scale, when clinically feasible. A psychiatric disturbance was recorded when diagnostic criteria were fulfilled according to the respective validated scale and documented in the medical record by the treating physician. Renal insufficiency was defined as an acute rise in serum creatinine above baseline values according to institutional laboratory reference ranges. Pressure ulcers were defined as skin lesions documented in medical records and confirmed by clinical examination. Neurological complications included hemorrhagic transformation and documented neurological deterioration, such as newly developed dysphagia or epileptic seizures, during hospitalization.

Laboratory assessments at admission included a basic metabolic panel (urea, creatinine, glucose, sodium, potassium, chloride), a complete blood count (leukocyte count, erythrocyte count, platelet count, hemoglobin, hematocrit), and a lipid panel (high-density lipoprotein (HDL), low-density lipoprotein (LDL), and triglycerides). Blood pressure values at admission were recorded, along with post-AIS complications. 

Final outcomes were categorized as survival, death, or discharge/transfer to another hospital ward, and the total length of hospital stay was recorded.

Statistical analysis

Statistical analysis was performed using SPSS, version 26 (IBM Corp., Armonk, NY).Continuous variables were assessed for normality using the Shapiro-Wilk test. Normally distributed variables are presented as mean ± standard deviation and were compared between groups using the Student’s t-test. Non-normally distributed variables are presented as median (interquartile range (IQR)) and were compared using the Mann-Whitney U test. Categorical variables are presented as counts and percentages and were compared using the chi-square test or Fisher’s exact test, as appropriate. All tests were two-tailed, and a *P*-value <0.05 was considered statistically significant. We acknowledge that multiple statistical tests were performed; however, no formal correction for multiple comparisons was applied due to the exploratory nature of the study.

## Results

A total of 150 patients with AIS were included in the analysis, of whom 73 developed complications during hospitalization (Group 1), and 77 did not (Group 2). Patients in Group 1 were older than those in Group 2 (median age 73.0 (IQR 66.5-79.0) vs. 69.0 (IQR 62.0-73.0) years, *P* < 0.001). There was no significant difference in gender distribution between the groups (*P* = 0.630). Regarding comorbidities, diabetes mellitus was more prevalent in Group 1 compared with Group 2 (67.1% vs. 16.9%, *P* = 0.023), and cardiological comorbidities were also more frequent among patients who developed complications (54.8% vs. 31.2%, *P* = 0.037). No significant between-group differences were observed for hypertension, renal, hepatic, rheumatological, or other comorbid conditions. Stroke severity at admission, assessed using the NIHSS score, was higher in patients who developed complications compared with those who did not. Both NIHSS measurements showed higher median values in Group 1 compared with Group 2 (NIHSS 1: 10.0 [5.0-16.0] vs. 5.0 (IQR 4.0-7.0), *P* < 0.001; NIHSS 2: 6.0 (IQR 4.0-11.0) vs. 3.0 (IQR 2.0-5.0), *P* < 0.001). Color Doppler duplex findings revealed no significant differences in the degree of carotid artery stenosis or vertebral artery pathology between the groups (*P* = 0.876). Brain CT imaging showed a higher proportion of large cerebral infarctions in patients who developed complications compared with those who did not (57.5% vs. 27.3%), whereas small infarctions were more frequent in patients without complications (72.7% vs. 42.5%, *P* < 0.001) (Table 1).

**Table 1:** Comparison of demographic data, comorbidities, NIHSS score, and brain CT findings between the groups Continuous variables are presented as median (interquartile range (IQR)) and compared between groups using the Mann-Whitney U test. Categorical variables are presented as the number of patients (*n*, %) and compared using the Pearson chi-square test. Test statistics (U or χ²) and corresponding *P*-values are shown in the respective columns. Statistical significance was defined as *P* < 0.05. For comorbidities, values represent the number and percentage of patients with the condition. Group 1 includes patients who developed complications, and Group 2 includes patients who did not develop complications. HTN, hypertension; DM, diabetes mellitus; NIHSS, National Institutes of Health Stroke Scale; CDD, color Doppler duplex; CT, computed tomography

Parameters		Group 1 (*N* = 73)	Group 2 (*N* = 77)	Test statistic	P
Age		73.00 (66.50-79.00)	69.00 (62.00-73.00)	*U* = 1800	0.000
Gender (*n*, %)					
Female		37 (50.70)	36(46.90)	*χ*² = 0.31	0.630
Male		37 (49.30)	41(53.20)		
Comorbidities (*n*, %)					
HTN		53 (72.60)	57 (74.02)	*χ*² = 0.00	1.000
DM		49 (67.10)	13 (16.90)	*χ*² = 37.7	0.023
Cardiological (coronary artery disease)		40 (54.80)	24 (31.20)	*χ*² = 8.34	0.037
Renal		20 (27.39)	25 (32.46)	*χ*² = 0.15	1.000
Hepatic		2 (2.73)	4 (5.19)	*χ*² = 0.52	0.887
Rheumatological		7 (9.58)	7 (9.09)	*χ*² = 0.52	0.966
Other		2 (2.73)	3 (3.89)	*χ*² = 1.24	0.273
NIHSS score					
NIHSS T1		10.00 (5.00- 16.00)	5.00 (4.00-7.00)	*U* = 1,550	0.000
NIHSS T2		6.00 (4.00-11.00)	3.00 (2.00-5.00)	*U *= 1,650	0.000
CDD (*n*, %)					
Carotid artery stenosis	Mild	39 (53.40)	42 (54.50)	*χ*² = 0.04	0.876
	Moderate	25 (34.20)	24 (31.20)		
	Severe	7 (9.60)	7 (9.10)		
Vertebral artery pathology		2 (2.70)	4 (5.20)		
Cerebral infarction on brain CT (*n*, %)					
Small		31 (42.50)	56 (72.70)	*χ*² = 0.00	0.000
Large		42 (57.50)	21 (27.30)		

No significant differences were observed between the groups in baseline hematological parameters, renal function tests, electrolyte levels, or lipid profiles. Admission glucose levels were higher in Group 1 compared with Group 2 (14.0 (IQR 10.1-16.3) vs. 6.8 (IQR 5.95-9.65) mmol/L, *P* = 0.027). Blood pressure categories did not differ significantly between the groups (*P* = 0.158). Among patients in Group 1, the most frequent in-hospital complications were urinary tract infections, pneumonia, and urinary and fecal incontinence (*P* = 0.001), followed by psychiatric complications, epileptic seizures, and hemorrhagic transformation (*P* = 0.003 each), renal insufficiency, decubitus ulcers, and pulmonary thromboembolism (*P* = 0.006 each), and dysphagia and deep vein thrombosis (*P* = 0.054 each). Clinical outcomes differed between the groups. Patients in Group 2 were more frequently discharged home, whereas in-hospital mortality was higher in Group 1 (17.8% vs. 3.9%, *P* = 0.017). Length of hospital stay was longer in Group 1 compared with Group 2 (14.0 (IQR 10.0-17.0) vs. 7.0 (IQR 6.0-10.0) days, *P* < 0.001) (Table 2).

**Table 2:** Comparison of laboratory, BP, complications, outcome, and length of hospital stay between the groups. Continuous variables are presented as median (interquartile range (IQR)) and compared between groups using the Mann-Whitney U test. Categorical variables are presented as the number of patients (*n*, %) and compared using the chi-square test or Fisher’s exact test when expected cell counts were <5. Test statistics (U or χ²) and corresponding *P*-values are reported in the respective columns. Statistical significance was defined as *P* < 0.05. Group 1 includes patients who developed complications, and Group 2 includes patients who did not develop complications. BP, blood pressure; HDL, high-density lipoprotein; LDL, low-density lipoprotein; UTI, urinary tract infection; DVT, deep vein thrombosis; PTE, pulmonary thromboembolism

Parameters	Group 1 (*N* = 73)	Group 2 (*N* = 77)	Test statistic		P
Leukocytes (×10⁹/L)	8.90 (8.11-9.54)	7.80 (6.90-9.90)	*U *= 2620		0.138
Erythrocytes (×10¹²/L)	4.70 (4.18-5.05)	4.59 (4.30-5.10)	*U *= 2800		0.997
Hematocrit (L/L)	4.20 (3.80-.4.50)	4.27 (3.90-4.40)	*U *= 2750		0.856
Hemoglobin (g/L)	139.00 (123.50-150.50)	140.00 (128.50-153.00)	*U *= 2700		0.709
Platelets (×10⁹/L)	237.00 (195.50-303.50)	220.00 (181.50-289.00)	*U *= 2600		0.141
Urea (mmol/L)	6.80 (5.35-9.00)	6.50 (5.20-9.00)	*U *= 2720		0.814
Creatinin (mmol/L)	75.00 (60.00-95.00)	79.00 (63.50-96.00)	*U *= 2850		0.469
Glucose (mmol/L)	14.00 (10.10-16.30)	6.80 (5.95-9.65)	*U *= 2100		0.027
Sodium (mmol/L)	139.00 (138.00-141.00)	139.00 (136.00-141.00)	*U *= 2480		0.080
Potassium (mmol/L)	4.00 (3.80-4.40)	4.10 (3.70-4.40)	*U *= 2780		0.942
Chlorides (mmol/L)	103.00 (100.00-105.50)	101.00 (98.50-104.00)	*U *= 2470		0.096
HDL (mmol/L)	1.00 (0.90-1.20)	1.10 (0.90-1.30)	*U *= 2650		0.477
LDL (mmol/L)	2.80 (2.00-3.60)	2.70 (2.20-3.65)	*U *= 2730		0.830
Triglycerides (mmol/L)	1.42 (1.20-2.16)	1.62 (1.30-2.24)	*U *= 2570		0.225
BP, mmHg (*n*, %)					
<130/80	14 (19.20)	14 (18.20)	χ²=4.2		0.158
130/80-139/89	8 (11.00)	19 (24.70)			
>140/90	32 (43.80)	30 (39.00)			
>180/120	19 (26.00)	14 (18.20)			
Complications (*n*, %)					
UTI	26 (35.00)	0.00 (0.00)	Fisher’s exact		0.001
Pneumonia	20 (27.00)	0.00 (0.00)	Fisher’s exact		0.001
Dysphagia	4 (5.00)	0.00 (0.00)	Fisher’s exact		0.054
Epileptic seizure	8 (11.00)	0.00 (0.00)	Fisher’s exact		0.003
Hemorrhagic transformation	8 (11.00)	0.00 (0.00)	Fisher’s exact		0.003
Renal insufficiency	7 (9.00)	0.00 (0.00)	Fisher’s exact		0.006
Urinary and fecal incontinence	12 (16.00)	0.00 (0.00)	Fisher’s exact		0.001
Psychiatric	8 (11.00)	0.00 (0.00)	Fisher’s exact		0.003
Decubitus	7 (9.00)	0.00 (0.00)	Fisher’s exact		0.006
DVT	4 (5.00)	0.00 (0.00)	Fisher’s exact		0.054
PTE	7 (9.00)	0.00 (0.00)	Fisher’s exact		0.006
Outcome (*n*, %)					
Discharge	58 (79.50)	73 (94.80)	χ²=6.0		0.017
Transfer to ward	1 (1.30)	2 (2.70)			
Death	13 (17.80)	3 (3.90)			
Length of hospital stay (days)	14.00 (10.00-17.00)	7.00 (6.00-10)	U=1650		0.000

## Discussion

This retrospective observational cohort study examined patients with first AIS, comparing those who developed in-hospital complications with those who did not. Patients who developed complications were older, had more severe strokes, larger infarctions, higher admission glucose levels, longer hospital stays, and higher in-hospital mortality. These observations describe differences between patients with and without complications and provide a descriptive overview of clinical and laboratory characteristics in this cohort.

In general, the frequency of in-hospital complications among hospitalized ischemic stroke patients ranges from approximately one-third to more than one-half [13]. In our study, 48% of patients experienced one or more in-hospital complications, which is lower than that reported in some previous studies [14]. The study by Langhorne et al. included patients with hemorrhagic stroke, which may partly explain this difference [15]. Patients who developed in-hospital complications had significantly higher NIHSS scores both at admission and at discharge. Approximately one third of patients with complications (31.5%) had a severe stroke on admission (NIHSS >15), compared with only 6% of patients without complications. Moderate stroke severity (NIHSS 5-15 at admission) was observed in 50.6% of patients with complications and in 57% of patients without complications. These findings are comparable to those reported in a similar study [16]. Overall, patients with in-hospital complications presented with more severe strokes, as reflected by both higher NIHSS scores and lower ASPECTS scores on CT imaging [17]. They also had longer hospital stays, poorer in-hospital outcomes, and significantly higher mortality. Infectious complications were the most common, particularly urinary tract infections and pneumonia, followed by urinary and fecal incontinence. Pneumonia in stroke patients is often related to dysphagia and aspiration, and older age, hypertension, and diabetes have been associated with an increased risk of dysphagia in patients with ischemic stroke [18]. In our study, patients who developed complications were significantly older and had a higher prevalence of cardiac disease and diabetes. Similarly, a large study by Vakilian et al, which included 2,199 patients with ischemic and hemorrhagic stroke, identified hypertension and diabetes mellitus as major risk factors among patients who died, with neurological and infectious complications being the most frequent fatal complications [19]. In a Nigerian study of patients with first-ever AIS, the presence of complications was the strongest predictor of short-term mortality [20]. Our results also demonstrated that patients with in-hospital complications had significantly higher admission blood glucose levels than those without complications. Civelek et al. [21] reported that the five most common post-stroke complications following a first ischemic stroke were urinary tract infection, shoulder pain, insomnia, depression, and other musculoskeletal pain. A previous study reported that in-hospital complications, particularly respiratory infections and cerebral edema, were independently associated with increased in-hospital mortality, with a stronger effect observed in patients with more severe strokes [22]. Similar findings were reported in a study conducted in Burkina Faso [23]. In patients with small subcortical infarctions, elevated blood glucose levels have been associated with greater stroke severity [24], and admission hyperglycemia has been linked to worse outcomes and an increased risk of in-hospital mortality in stroke patients [25]. These findings may help identify patients at higher risk for in-hospital complications and support early preventive strategies. Compared with earlier studies, the present study focuses specifically on the clinical characteristics of patients with ischemic stroke who develop in-hospital complications, allowing for a clearer comparison with patients without complications and enabling identification of potential measures to reduce complication rates. Many previous studies included both ischemic and hemorrhagic stroke, patients with recurrent stroke or other cerebrovascular events, or did not differentiate between patients with and without complications, making it more difficult to conclude specific clinical and laboratory predictors.

This study has several limitations. The retrospective design of our study limited the availability of certain laboratory parameters, such as D-dimer and inflammatory markers, which were not routinely measured in all patients with AIS. While these markers do not directly cause ischemic stroke, they could have provided additional insight into thrombotic and inflammatory processes that may contribute to in-hospital complications, such as infections or thromboembolic events. Their absence may have restricted a more detailed analysis of these specific risk factors. Additionally, the single-center nature of the study and the relatively small sample size may affect the generalizability of the findings. Despite these limitations, the results provide valuable insights and serve as a basis for future prospective, multicenter studies aimed at further exploring the clinical and laboratory predictors of complications in patients with AIS.

## Conclusions

In this study, we identified several clinically relevant factors associated with the occurrence of in-hospital complications among patients with first-ever AIS. Patients who developed complications during hospitalization were more likely to be older, have greater stroke severity at presentation, have elevated blood glucose levels on admission, and have a history of cardiac comorbidities or diabetes. Together, these findings highlight the multifactorial nature of early stroke-related complications and underscore the importance of comprehensive early assessment. Recognizing high-risk patients at admission may facilitate closer monitoring and timely, individualized management, which could contribute to improved in-hospital care and outcomes for this vulnerable patient population.
